# A Manual of Surgery

**Published:** 1886-12

**Authors:** 


A Manual of Surgery. *In Treatises by Various Authors. In three volumes. By* Frederick Treves, F.R.C.S., *Surgeon to and Lecturer on Anatomy at the London Hospital. Vol. I., General Surgical Affections, The Blood-vessels; The Nerves; The Skin. Vol. II., The Thorax; The Organs of Digestion; The Genito-Urinary Organs. Vol. III., The Organs of Locomotion and of Special Sense; The Respiratory Passages; The Head; The Spine. Duodecimo, 1866 pages, 213 engravings. Philadelphia:* Lea Brothers & Company, *1886. Chicago:* A. C. McClurg & Company.

This book is a successful attempt to represent the principles and practice of modern surgery in the form and manner most acceptable to the greatest number of practitioners and medical students. It is "Concerned mainly with the Clinical, Diagnostic and Therapeutic Aspects of Surgery." Thirty-three hospital surgeons of Great Britain and Ireland are the "Various Authors" of the fifty-nine treatises" or monographs of which the "Manual" is composed.

The name of Frederick Treves as editor, who himself writes some of the most important articles, is sufficient guarantee that the work is all that it claims to be. Besides the editor, many of the contributors are also well known writers and clinical teachers, while we find a number of new names in the list. All the articles are of a high order of merit. There seems to have been a special effort throughout to incorporate and emphasize recently established doctrines and methods.

There are not many illustrations, but what there are are mostly original, and always to the point. The "Manual" is destined to become popular both as a text-book and as a book of reference, and to take rank with the standard works on surgery.

